# Magma Seawater Inhibits Hepatic Lipid Accumulation through Suppression of Lipogenic Enzymes Regulated by SREBPs in Thioacetamide-Injected Rats

**DOI:** 10.3390/md17060317

**Published:** 2019-05-30

**Authors:** Minji Woo, Jeong Sook Noh, Mi Jeong Kim, Yeong Ok Song, Hyunjoo Lee

**Affiliations:** 1 Department of Food Science and Nutrition, Kimchi Research Institute, Pusan National University, Busan 46241, Korea; woo07140@pusan.ac.kr (M.W.); yosong@pusan.ac.kr (Y.O.S.); 2 Division of Functional Food Research, Korea Food Research Institute, 245 Nongsaengmyeong-ro, Iseo-myeon, Wanju-gun, Jeollabuk-do 55365, Korea; 3 Department of Food Science and Nutrition, Tongmyong University, Busan 48520, Korea; jsnoh2013@tu.ac.kr; 4 Department of Food and Nutrition, Silla University, Busan 46958, Korea; mjkim@silla.ac.kr; 5 Wellness Life Institute Co., Ltd., Jeju 63246, Korea

**Keywords:** magma seawater, thioacetamide, lipid metabolism, oxidative stress, antioxidant enzymes

## Abstract

Thioacetamide (TAA) is known to induce lipid accumulation in the liver. In the present study, we investigated the effects of magma seawater (MS) rich in minerals on hepatic lipid metabolism by evaluating lipogenic enzymes regulated by sterol regulatory element-binding proteins (SREBPs). Rats (*n* = 10 per group) were intraperitoneally injected with TAA (200 mg/kg bw) thrice a week for seven weeks in combination with a respective experimental diet. Rats in the TAA-treated group received either a chow diet (Control group) or a chow diet containing MS (TMS group, 2.05%) or silymarin (TSM group, 0.05%). Rats in the normal group were injected with PBS as a vehicle and received a chow diet. Rats in the TMS group showed significantly lower hepatic lipid concentrations than rats in the control group (*p* < 0.05). Hepatic protein expression levels of fatty acid synthase, SREBP-1, 3-hydroxy-3-methylglutaryl-coenzyme A reductase, and SREBP-2 were significantly downregulated in the TMS group, whereas carnitine palmitoyltransferase 1 levels were upregulated (*p* < 0.05). Hepatic thiobarbituric acid reactive substances levels were lower in the TMS group, whereas protein levels of glutathione peroxidase and catalase were elevated (*p* < 0.05). The effects of MS were comparable to those of silymarin. Our results evidently showed that MS inhibits hepatic lipid accumulation by suppressing lipid synthesis, accompanied by lipid oxidation and elevation of antioxidative status.

## 1. Introduction

The health-promoting effects of deep seawater have been studied in terms of antioxidative [1], anti-inflammatory [2], anti-obesity [3], and anti-diabetic effects [4]. Magma seawater is underground seawater that is naturally filtered by basaltic rocks in Jeju Island, Korea. The characteristics of magma seawater are similar to those of deep seawater but is more easily accessible because it is found between 100 and 200 m below sea level [5]. Magma seawater contains sodium (Na), magnesium (Mg), calcium (Ca), potassium (K), iron (Fe), zinc (Zn), selenium (Se), vanadium (V), manganese (Mn), and germanium (Ge) [6]. The health benefits of trace minerals are well established. The antioxidant effects of V were demonstrated to exert health benefits against obesity and diabetes [7,8]. Ge also possesses antioxidative property [9] and suppresses tumor progression [10]. In addition, beneficial effects of Se, Ge, Sn, Mg, and Ca on liver function have been reported [11,12,13,14].

The liver is a vital organ that performs multiple metabolic functions, such as metabolism of nutrients, bile secretion, and detoxification [15]. Hepatic steatosis is the primary cause of the development of degenerative liver diseases. Thioacetamide (TAA), a hepatotoxin, could lead to the development of steatosis in the liver because TAA metabolites alter cell permeability and inhibit mitochondrial activity [16,17], which in turn impair the synthesis and secretion of lipoproteins [18]. Cytochrome P450 enzymes in the liver sequentially metabolize TAA to TAA-S-oxide and TAA-S-dioxide, which are highly toxic to hepatocytes. Long-term use of TAA can damage the liver cells, which subsequently leads to acute liver failure or hepatic cirrhosis [19]. In addition, oxidative stress could be elevated during the detoxification of TAA because mixed function oxidases participate in this metabolic process [16]. Oxidative stress caused by TAA enhances lipid peroxidation in the liver as indicated by the increased level of thiobarbituric acid reactive substances (TBARS) [20].

Lipid metabolism is tightly regulated by several transcription factors. During lipogenesis, sterol regulatory element-binding protein (SREBP)-1 and -2 regulate fatty acid synthase (FAS) and 3-hydroxy-3-methylglutaryl-coenzyme A reductase (HMGCR), respectively [21]. By contrast, during lipolysis, enzymes such as carnitine palmitoyltransferase 1 (CPT1) promote fatty acid oxidation by translocating the long chain fatty acid to the mitochondria. In particular, oxidative stress upregulates the expression of SREBPs [22]. Recent studies have focused on antioxidative functional substances to improve liver diseases by attenuating oxidative stress [23,24]. In the present study, the beneficial effects of magma seawater on hepatic lipid metabolism were investigated in TAA-injected rats with respect to prevention of lipid accumulation and elevation of antioxidative status and the corresponding mechanisms of action were explored.

## 2. Results

### 2.1. Changes in Body Weight, Food Efficiency, and Liver Weights

**Figure 1:**
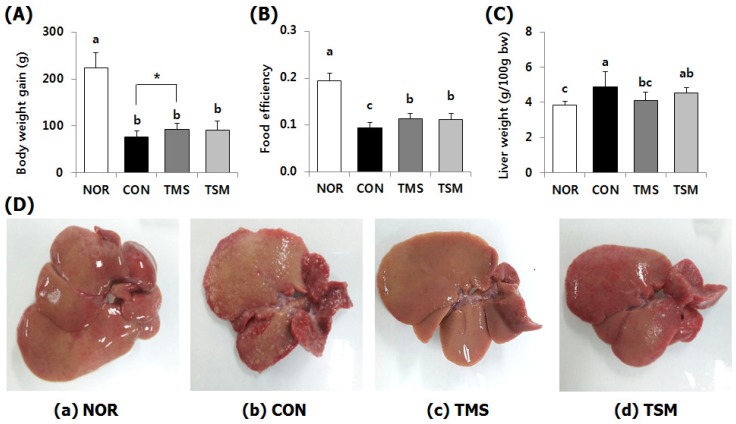
Effect of magma seawater on the body weight gain, food efficiency, liver weight, and macroscopic appearance of liver of thioacetamide-injected rats. Data are mean ± SD (*n* = 10 in each group). (**A**) Body weight gain (g); (**B**) Food efficiency was calculated as body weight gain (g)/total food intake (g); (**C**) Liver weight (g/100 g of body weight); (**D**) Macroscopic appearance of liver; Normal (NOR) group, Spraque–Dawley (SD) rat fed chow diet and injected phosphate buffered saline intraperitoneally three times a week for seven weeks; Control (CON) group, SD rat fed chow diet and injected thioacetamide (TAA) (200 mg/kg bw) intraperitoneally three times a week for seven weeks; TMS group, SD rat fed chow containing 2.05% (*w*/*w*) of magma seawater powder and injected TAA as the same manner as the CON group; TSM group, SD rat fed chow diet containing 0.05% (*w*/*w*) of silymarin and injected TAA as the same manner as the CON group. ^a–c^ Data with different letters in the column are significantly different with one-way ANOVA followed by Duncan’s multiple range test at *p* < 0.05. * Significantly different between CON and TMS groups by student’s *t*-test (*p* = 0.034).

The body weight gains of rats in the only TAA-injected control group (CON group) were significantly lower than those of rats in the normal group (NOR group), indicating that TAA significantly induced hepatotoxicity (Figure 1A). However, the body weight gains of rats in the TAA-injected magma seawater group (TMS group) and TAA-injected silymarin group (TSM group) were slightly higher. The CON and TMS groups showed a significant difference in weight gains (*p* < 0.05, *t*-test). The food efficacies of rats in the TMS and TSM groups were significantly higher compared to those of rats in the CON group (*p* < 0.05) (Figure 1B). Liver weights of rats in the TMS group were significantly lower than those of rats in the CON group (*p* < 0.05) (Figure 1C).

### 2.2. Macroscopic Examination of Liver

Compared with the appearance of NOR group (Figure 1a), the surfaces of the livers of rats in the CON group contained many small lumps (Figure 1b), indicating that TAA induced liver injury. However, the livers of rats in the TMS (Figure 1c) and TSM (Figure 1d) groups were relatively smoother compared to the livers of rats in the CON group, indicating reduced hepatic steatosis.

### 2.3. Lipid Concentrations in the Liver

**Figure 2:**
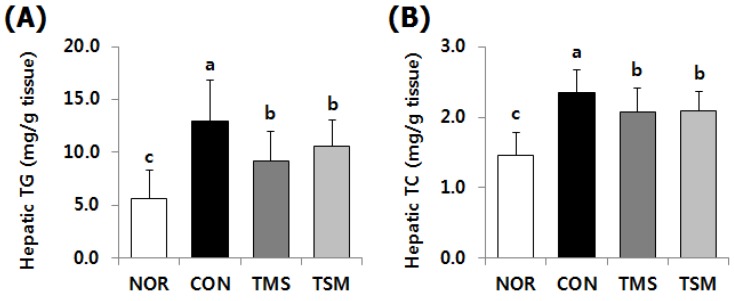
Effect of magma seawater on hepatic lipid levels of thioacetamide-injected rats. Data are mean ± SD (*n* = 10 in each group). (**A**) Hepatic triglyceride (TG) level; (**B**) Hepatic total cholesterol (TC) level. See the legend of Figure 1 for experimental groups in detail. ^a–c^ Data with different letters are significantly different with one-way ANOVA followed by Duncan’s multiple range test at *p* < 0.05.

Hepatic triglyceride (TG) and total cholesterol (TC) levels in the TAA-injected groups were significantly higher than those in the NOR group (Figure 2). However, hepatic TG and TC levels in the TMS group were 28.9% and 11.9% lower compared to those in the CON group, respectively (*p* < 0.05), while those in the TSM group were 18.3% and 11.5% lower, respectively (*p* < 0.05).

### 2.4. TBARS and GSH/GSSG Levels

TBARS levels were significantly higher in the TAA-injected groups than those in the NOR group (Table 1, *p* < 0.05). By contrast, TBARS levels in the TMS and TSM groups were significantly lower by 22.1% and 23.8%, respectively, compared to those in the CON group (*p* < 0.05). Hepatic glutathione (GSH) levels were significantly higher in the TAA-injected groups than those in the NOR group (*p* < 0.05), implying that a compensatory mechanism occurred in the TAA-injected group because of higher hepatotoxin levels. The TAA-injected groups showed no significant differences in GSH levels. However, glutathione disulfide (GSSG) levels in the TMS and TSM groups were significantly lower by 31.2% and 35.0%, respectively, compared to those in the CON group (*p* < 0.05). Therefore, the GSH/GSSG ratios in the TMS and TSM groups were higher by 138.6% and 154.4%, respectively, compared to those in the CON group (*p* < 0.05).

### 2.5. Western Blot Results

#### 2.5.1. Expression of Antioxidative Enzymes

**Figure 3:**
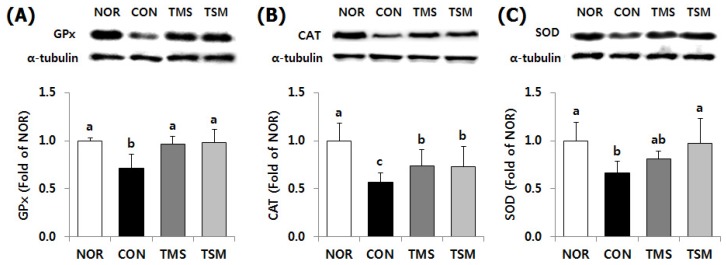
Protein expression level for antioxidant enzymes in the liver of thioacetamide-injected rats. Data are mean ± SD (*n* = 10 in each group). (**A**) GPx, glutathione peroxidase; (**B**) CAT, catalase; (**C**) SOD, superoxide dismutases. See the legend of Figure 1 for experimental groups in detail. ^a–c^ Data with different letters are significantly different with one-way ANOVA followed by Duncan’s multiple range test at *p* < 0.05.

Protein expression levels of glutathione peroxidase-1/2 (GPx) and catalase (CAT) were higher in the TMS group by 135.5% and 129.9%, relative to those in the CON group, respectively. GPx and CAT levels in the TMS group were higher by 137.4% and 129.1%, respectively (Figure 3, *p* < 0.05). The protein level of superoxide dismutases-1 (SOD) in the TSM group was significantly higher by 146.3% compared to that in the CON group (*p* < 0.05). The TMS group showed higher protein levels of SOD, although the observed difference was not statistically significant.

#### 2.5.2. Expression of Lipid Metabolism-Related Factors

**Figure 4:**
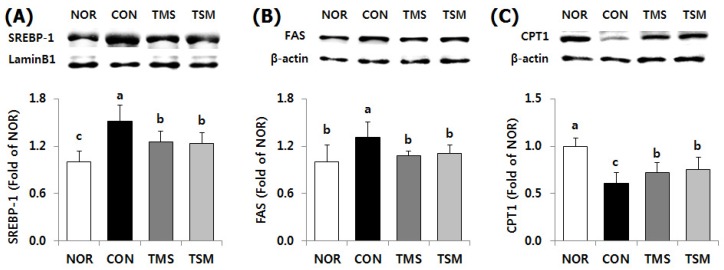
Protein expression level for fatty acid synthesis and oxidation in the liver of thioacetamide-injected rats. Data are mean ± SD (*n* = 10 in each group). (**A**) SREBP-1, sterol regulatory element-binding protein-1; (**B**) FAS, fatty acid synthase; (**C**) CPT1, carnitine palmitoyltransferase 1. See the legend of Figure 1 for experimental groups in detail. ^a–c^ Data with different letters are significantly different with one-way ANOVA followed by Duncan’s multiple range test at *p* < 0.05.

Protein expression levels of SREBP-1, a transcription factor for fatty acid synthesis, were significantly downregulated in the TMS and TSM groups by 17.7% and 18.5%, respectively, compared to those in the CON group (Figure 4, *p* < 0.05). As expected, FAS protein levels were significantly lower in the TMS and TSM groups by 17.8% and 15.3%, respectively (*p* < 0.05). By contrast, CPT1 protein levels were significantly higher in the TMS and TSM groups by 119.4% and 124.1%, respectively (*p* < 0.05).

**Figure 5:**
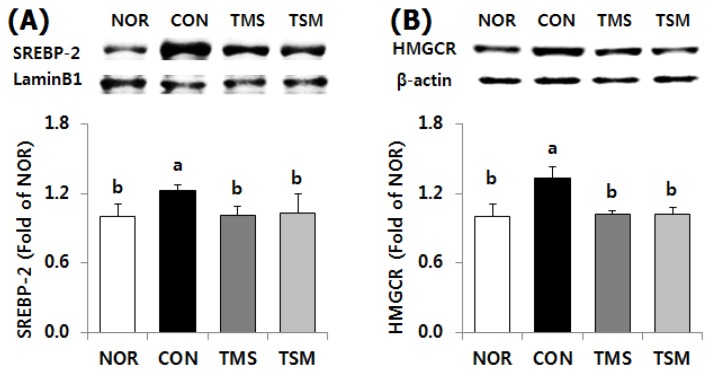
Protein expression level for cholesterol synthesis in the liver of thioacetamide-injected rats. Data are mean ± SD (*n* = 10 in each group). (**A**) SREBP-2, sterol regulatory element-binding protein-2; (**B**) HMGCR, 3-hydroxy-3-methylglutaryl-coenzyme A reductase. See the legend of Figure 1 for experimental groups in detail. ^a,b^ Data with different letters are significantly different with one-way ANOVA followed by Duncan’s multiple range test at *p* < 0.05.

The expression levels of SREBP-2, a transcription factor for cholesterol synthesis, were significantly downregulated in the TMS and TSM groups by 17.6% and 15.7%, respectively, compared to those in the CON group (Figure 5, *p* < 0.05). HMGCR protein levels in the TMS and TSM groups were significantly lower by 23.4% and 23.5%, respectively, compared to those in the CON group (*p* < 0.05).

## 3. Discussion

Magma seawater, which is filtered through basaltic rocks, has been recognized as a new source of mineral water that is free from contaminants and is valued for its high mineral content, similar to deep seawater. Magma seawater has the advantage of availability because it is located between 100 and 200 m below sea level [5]. In our previous study, we demonstrated the protective effects of magma seawater against TAA induced hepatotoxicity in the liver via upregulation of phase I and II enzymes in the detoxification process and the upregulation of antioxidant enzymes [6]. These effects could be attributed to the minerals such as Na, Mg, Ca, V, Fe, Zn, Se, V, and Ge present in magma seawater, which contains 9987 mg/L [6]. In the present study, we investigated whether magma seawater can exert lipid-lowering effects and elucidated their corresponding mechanisms of action because failure of hepatic lipid metabolism is considered a primary cause of liver diseases [15]. Our results showed that magma seawater exerted inhibitory effects on hepatic lipid accumulation by suppressing fat synthesis with a concomitant increase in fat oxidation in TAA-injected rats. Subsequently, magma seawater suppressed lipid peroxidation, which is also known to induce the expression of SBEBPs by increasing oxidative stress [16,22]. 

Lipid metabolism is tightly regulated by several transcription factors. SREBPs are the most important transcription factors that induce the expression of genes involved in fatty acid and cholesterol synthesis, including FAS and HMGCR, in the liver [21]. In the present study, supplementation with magma seawater, which is rich in minerals, downregulated the expression of SREBP-1 and -2, which in turn inhibited the expression of the lipogenic enzymes FAS and HMGCR. By contrast, expression levels of CPT1, which is responsible for the translocation of long-chain fatty acids into the mitochondria matrix, were found to be upregulated. The inhibition of lipogenesis and enhancement of lipid oxidation by magma seawater led to reduced TG and TC concentrations in the livers of rats injected with TAA. These observed effects of the magma seawater were comparable to those of silymarin. Few studies have demonstrated the inhibitory effects of seawater on hepatic lipid accumulation. The underground seawater (Jeju Island, Korea) reduced hepatic TG via downregulation of FAS in high-fat diet-fed mice [25]. Similarly, deep seawater reduced hepatic TG and TC levels and decreased liver size in high-fat diet-fed hamsters [3]. These previous findings are in good agreement with the results of our study. Moreover, the hepatic lipid-lowering effects of minerals have been extensively studied. Se-enriched probiotics are known to improve hepatic lipid metabolism mediated by the upregulation of CPT1 expression and downregulation of SREBP-1 and FAS expression [26], whereas Se deficiency increased hepatic HMGCR activity [27]. In addition, Mg-rich deep seawater promoted the fecal excretion of lipids, leading to reduced hepatic lipid levels [4]. Low dietary Ca levels were positively associated with fat synthesis [28]. Mg and Ca supplementation are recommended to lower body weight and fat levels [29,30]. Mg and Ca were found to be the most abundant anions present in the magma seawater used in the current study, with corresponding concentrations of 1173 and 297 mg/L, respectively, which accounted for 12% and 3% of the total mineral content (9987 mg/L) [6]. Nevertheless, the protective effects of minerals from Se, Ge, Sn, Mg, and Ca in the liver are well established [11,12,13,14]. In addition, the plasma lipid-lowering effects of minerals including Se [31] and Zn [32,33] have been studied. Taken together, the above findings and our current findings showed that magma seawater, which is rich in minerals, exerts lipid-lowering effects. 

TAA is metabolized to TAA-S-oxide and TAA-S-dioxide by cytochrome P450 during the detoxification process. These metabolites are highly toxic and interfere with mitochondrial activity by altering the cell permeability [16,17], which in turn impairs the synthesis and secretion of lipoproteins [18] thereby lead to lipid accumulation. As discussed earlier, hepatic lipid concentrations in the magma seawater-supplemented rats were lower even when the rats were administered with TAA, with the assumption that TAA might be readily metabolized and excreted. This phenomenon was confirmed in our previous study. The protein levels of phase I and II detoxification enzymes such as cytochrome P450 2E1, glutathione S-transferase, and methionine adenosyltransferase were found to be significantly elevated by magma seawater supplementation [6]. As a result, the liver damage by TAA injection was attenuated in magma seawater-fed groups as evidenced by reduction of plasma aminotransferase activities and histological analysis such as Masson’s trichrome and H&E staining [6]. In addition, our findings indicated that magma seawater supplementation attenuated hepatic steatosis induced by TAA. The liver surfaces of TAA-injected rats were covered with small lumps that reflect areas of lipid accumulation (Figure 1b), whereas magma seawater (Figure 1c) or silymarin supplementation (Figure 1d) appeared to remove the small lumps, indicating that magma seawater exerts inhibitory effects on lipid accumulation. Steatohepatitis induced by TAA is similar to nonalcoholic steatohepatitis observed in humans. These effects were comparable to those of silymarin, a known antioxidant. 

Oxidative stress is a well-known pathophysiological condition responsible for the development of numerous degenerative diseases, including liver disease. Antioxidants or functional molecules with antioxidative properties or compounds, including minerals that can elevate antioxidative status, exhibit great potential to improve diseases by ameliorating oxidative stress [23]. Previous studies suggested that liver damage induced by TAA is associated with the exacerbation of lipid peroxidation and depletion of antioxidant status [34]. In the present study, the TBARS and GSSG concentrations were elevated by TAA injection but were reduced by the magma seawater supplementation. In addition, hepatic protein levels of antioxidant enzymes were higher in magma seawater-supplemented rats. Our findings were consistent with those of previous studies, which showed that deep seawater reduced hepatic malondialdehyde (MDA) concentrations and maintained higher liver GSH and GPx levels [3,35]. In addition, underground seawater supplementation was found to increase CAT activity in HepG2 cells [25]. Furthermore, the antioxidative activities of minerals have been extensively studied. Mg and Se act as coenzymes of SOD and GPx [36,37]. Se supplementation is associated with higher hepatic concentrations of GSH and GPx and an increase the GSH/GSSG ratio in hamsters [38]. Zinc is a key mineral involved in antioxidant defense by acting as a cofactor of the SOD enzyme to modulate GSH metabolism and facilitate the reduction of free radicals [39]. In addition, V-fortified drinking water increased SOD, CAT, and GPx levels in liver cells [7]. Similarly, mineral-rich black vinegar containing K, Mg, Ca, Fe, Mn, and Se reduced hepatic TBARS levels but elevated GSH, GPx, and catalase activities [40]. The antioxidative activities of minerals are known to inhibit lipid peroxidation [38,41]. Oxidative stress has been established to contribute to increasing the activities of SREBPs [16,22], which in turn increase FAS and HMGCR levels *in vitro* and *in vivo* [42,43]. 

In the present study, magma seawater improved hepatic lipid metabolism via downregulation of SREBPs, FAS, and HMGCR whereas upregulation of CPT1. These regulatory effects were directly associated with reduced lipid concentrations in the liver. In addition, antioxidative status was increased by the upregulation of antioxidant enzymes. Long-term intake of magma seawater is expected to prevent liver disease, similar to other types of mineral water. Advanced human studies on magma seawater are required to confirm the results from the animal studies.

## 4. Materials and Methods

### 4.1. Preparation of Experimental Diet

Magma seawater attained from Han-dong (Jeju Island, Korea) was electro-dialyzed (AC-10-100; Changjo Techno, Seoul, Korea) according to the method previously described [6]. Briefly, magma seawater was desalinated under the condition of 12 mS/cm conductivity followed by freeze-drying. The mineral composition of desalinated magma seawater was as follow: Cl 5132, SO_4_^2−^ 1341, Mg 1173, Ca 297, K 1405, Na 604, Br 11, Sr 10.1, Si 9.38, B 3.46, F 1, Zn 0.013, Cu 0.008, Mo 0.003, V 0.04, Se 0.02, Fe < 0.01, Mn 0.007, and Ge < 0.001 mg/mL [6]. The total concentration of magma seawater was 9987 mg/L [6]. The amount of desalinated magma seawater powder added to the chow diet (Harlan Teklad Global 18% Protein Rodent Diet, #2018S, Harlan Teklad, Madison, WI, USA) was determined based on the daily water consumption of the rats (20–45 mL) [44]. To evaluate the beneficial effects of magma seawater, the maximum water consumption of 45 mL was selected for the study. A daily diet consumption of rat ranges from 15–30 g, with an average amount of 22 g. The portion of magma seawater in the diet was calculated as follows: 45 mL × 9987 mg/L of magma seawater ÷ 22 g/day = 2.05%. For the positive control, silymarin was added to the diet as 0.05% (*w*/*w*) [45]. Silymarin is a flavonoid compound with antioxidative effects and it is used as a medicinal drug for hepato-protection [45].

### 4.2. Experimental Groups and Treatment

Sprague–Dawley rats (male, six weeks old) were purchased from Dooyeol Biotech (Seoul, Korea). After one week of acclimatization, rats weighing 175.7–178.0 g were divided into four groups based on their body weight (*n* = 10 per group). Except for the NOR group, the rats were intraperitoneally injected with TAA (200 mg/kg bw) thrice a week for seven weeks in combination with a respective experimental diet. TAA-treated rats received either a chow diet (Harlan Teklad Global 18% Protein Rodent Diet #2018S; Harlan Teklad, Madison, WI, USA) (CON group), chow diet containing magma seawater (TMS group, 2.05%), or silymarin (TSM group, 0.05%). The NOR group rats were injected with phosphate buffered saline (PBS) as a vehicle and received the chow diet. Rats had free access to the diet and water. To evaluate food efficiency, initial and final body weights were measured, and the daily dietary intake was checked. After 12 h of fasting, rats were anesthetized by intraperitoneal administration of 50 mg/kg of zoletil 50 (Virbac Laboratories, Carros, France) and 5 mg/kg of xylazine (Bayer Korea, Seoul, Korea). The liver was excised after perfusion with ice-cold PBS (10 mM, pH 7.2). The liver was subjected to examination for the macroscopic appearance. Liver weight data were calculated as follows: liver weight (g)/body weight (g) × 100. The animal study was approved by the Pusan National University-Institutional Animal Care and Use Committee (PNU-IACUC, Approval Number: PNU-2014-0505).

### 4.3. Measurement of Thiobarbituric Acid-Related Substances and Glutathione

Liver homogenates were prepared with PBS (1:9, *w*/*v*) using an Ultra-Turrax tissue homogenizer (Janke & Kunkel, IKA®-Labortechnik, Saufen, Germany). TBARS concentrations were determined using a MDA as the standard [46]. GSH/GSSG concentrations were measured using a commercially available kit (G257-10, Dojindo, Kumamoto, Japan).

### 4.4. Hepatic Lipid Measurement

Hepatic lipids were extracted from the liver homogenate using chloroform:methanol (2:1, *v*/*v*) solvent according to a previously described method [47]. Hepatic TG (AM157S-K) and TC (AM202-K) concentrations were measured using a commercially available kit (Asan Pharmaceutical Co., Seoul, Korea).

### 4.5. Western Blot Analysis

Protein expression levels of genes involved in the lipid synthesis and antioxidant enzymes were determined by western blot analysis as previously described [48]. Nuclear fractions were used for the determination of SREBP-1 and -2, and cytosol fraction was used for the rest experiments. Briefly, sodium dodecyl sulfate-polyacrylamide gel electrophoresis was performed, and the bands were transferred to a nitrocellulose membrane. Protein expression was visualized using the enhanced chemiluminescence, equipped with CAS-400 (Core Bio, Seoul, Korea) and evaluated using ImageJ software (National Institutes of Health, Bethesda, MD, USA). The protein expression level was expressed as the ratio to α-tubulin, β-actin, or lamin B1. Primary antibody α-tubulin (ab52866), β-actin (ab8226), and FAS (ab22759) were purchased from Abcam Inc. (Cambridge, MA, USA). SOD (FL-154, sc-11407), CAT (F-17, sc-34285), GPx (B-6, sc-133160), SREBP-1 (H-160, sc-8984), SREBP-2 (H-164, sc-5603), HMGCR (H-300, sc-33827), CPT1 (sc-139482), and lamin B1 (ZL-5; sc-56145) were purchased from Santa Cruz Biotechnology Inc. (Santa Cruz, CA, USA).

### 4.6. Statistical Analysis

Values are presented as the mean ± standard deviation (SD). Statistical analyses were performed using SPSS version 20 (SPSS Inc., Chicago, IL, USA). Data were analyzed by One-way analysis of variance followed by Duncan’s multiple-range test as a post hoc analysis. To compare the effect of each group with that of the CON group, the data were analyzed using Student’s *t*-test. Differences were considered significant at *p* < 0.05.

## Figures and Tables

**Table 1:** Effect of magma seawater on hepatic thiobarbituric acid reactive substances and glutathione/glutathione disulfide levels of thioacetamide-injected rats.

Group	TBARS (nmol/g Tissue)	GSH (μmol/g Tissue)	GSSG (μmol/g Tissue)	GSH/GSSG Ratio
NOR	24.89 ± 6.17 ^c^	2.98 ± 0.96 ^b^	0.13 ± 0.04 ^a^	23.65 ± 6.36 ^c^
CON	50.00 ± 8.51 ^a^	6.52 ± 0.76 ^a^	0.13 ± 0.06 ^a^	53.08 ± 14.43 ^b^
TMS	37.85 ± 6.64 ^b^	7.05 ± 1.68 ^a^	0.09 ± 0.02 ^b^	73.55 ± 21.18 ^a^
TSM	40.36 ± 6.94 ^b^	7.16 ± 1.14 ^a^	0.09 ± 0.02 ^b^	81.95 ± 16.49 ^a^

Data are mean ± SD (*n* = 10 in each group). Normal (NOR) group, Spraque–Dawley (SD) rat fed chow diet and injected phosphate buffered saline intraperitoneally three times a week for seven weeks; Control (CON) group, SD rat fed chow diet and injected thioacetamide (TAA) (200 mg/kg bw) intraperitoneally three times a week for seven weeks; TMS group, SD rat fed chow containing 2.05% (*w*/*w*) of magma seawater powder and injected TAA as the same manner as the CON group; TSM group, SD rat fed chow diet containing 0.05% (*w*/*w*) of silymarin and injected TAA as the same manner as the CON group. ^a–c^ Data with different letters in the column are significantly different with one-way ANOVA followed by Duncan’s multiple range test at *p* < 0.05. TBARS, thiobarbituric acid-related substances; GSH, glutathione; and GSSG, glutathione disulfide.
